# Preventive effects of whitening mouthrinses against coffee-induced discoloration in different types of resin composites: a daily-use in-vitro study

**DOI:** 10.1007/s10266-026-01329-z

**Published:** 2026-02-02

**Authors:** Gülşah Aşik, Fatmanur Ari, Abdulkadir Özçinar, Mehmet Buldur

**Affiliations:** 1 Faculty of Dentistry, Çanakkale Onsekiz Mart University, Çanakkale, Türkiye; 2 Department of Restorative Dentistry, Faculty of Dentistry, Çanakkale Onsekiz Mart University, Çanakkale, Türkiye

**Keywords:** Resin composites, Mouthwashes, Color stability, Hydrogen peroxide, Optical properties

## Abstract

This in vitro study aimed to evaluate the preventive effects of whitening mouthrinses against coffee-induced discoloration and to assess their influence on the optical properties—color difference (Δ*E*_00_), translucency parameter (ΔTP), and whiteness index difference (ΔWID)—of different resin composite materials. Three resin composites were tested: Filtek One Bulk Fill (FOB), Charisma Diamond One (CDO), and Estellite Sigma Quick (ESQ). Specimens were immersed daily in coffee, followed by exposure to three whitening mouthrinses—Listerine Advanced White (LAW), Colgate Optic White (COW), and R.O.C.S. Whitening Mouthrinse (ROC)—for 3 months. Distilled water served as the negative control. Color measurements were conducted with a spectrophotometer on standardized black, white, and gray backgrounds. Δ*E*_00_ values were analyzed using mixed-design ANOVA or Kruskal–Wallis tests depending on data distribution, while ΔTP and ΔWID values were evaluated using appropriate non-parametric tests (*p* < 0.05). All whitening mouthrinses significantly reduced coffee-induced discoloration (*p* < 0.05). LAW and COW, both containing hydrogen peroxide, exhibited the lowest Δ*E*_00_ values (≈ 2.0–2.3), while ROC showed limited efficacy. Whitening mouthrinses did not significantly affect translucency (ΔTP) values (*p* > 0.05), whereas coffee exposure resulted in a statistically significant reduction in translucency. Whitening rinses produced a mild increase in whiteness (ΔWID ≈ + 1.0– + 1.6), below the clinical perceptibility threshold (ΔWID ≥  + 2.0). Among materials, the nanohybrid composite ESQ demonstrated the most stable optical behavior across all parameters. Hydrogen peroxide–containing whitening mouthrinses exerted a preventive effect against coffee-induced discoloration without producing clinically perceptible whitening, thereby helping to maintain the optical stability of composite restorations.

## Introduction

Discoloration of resin composite restorations due to daily consumption of staining beverages such as coffee is a frequently reported clinical concern in esthetic dentistry. The color-stabilized optical behavior of resin composite materials is influenced by both intrinsic and extrinsic factors. Intrinsic factors include the chemical composition of the resin matrix, filler content, particle size and morphology, and the refractive index compatibility between filler particles and the resin matrix, which collectively affect light scattering and optical stability [1]. Extrinsic factors mainly involve exposure to chromogenic agents such as coffee and other staining beverages, leading to pigment adsorption and absorption within the resin matrix [2]. In addition, interactions with oral hygiene and whitening products may further modify surface characteristics and optical behavior depending on their chemical composition and mechanism of action. With the increasing demand for aesthetics, not only the mechanical properties but also the long-term preservation of color stability and optical performance of restorative materials have gained great importance in dentistry. Nowadays, resin composites are widely preferred due to their esthetic and functional properties that closely mimic natural tooth structure; however, they tend to undergo discoloration over time as a result of exposure to extrinsic pigments such as coffee, tea, red wine, and tobacco [3–5]. The discoloration process is associated with mechanisms such as pigment diffusion into the resin matrix and adsorption of organic compounds on the surface. Factors including the type of resin matrix, refractive index mismatch between filler particles, and surface roughness directly influence color stability [6, 7]. Moreover, different composite systems may exhibit distinct optical behaviors depending on their filler structure, particle size and distribution, and monomer composition [8].

In esthetic restorative dentistry, the objective evaluation of optical properties is essential for assessing the long-term esthetic stability of composite restorations. Parameters such as color difference (Δ*E*_00_), translucency parameter (ΔTP), and whiteness index difference (ΔWID) are widely used to quantitatively assess changes in color, light transmittance, and whiteness of resin composites following staining or whitening challenges [8–10]. These parameters are influenced by material-related factors such as filler characteristics and resin matrix composition, and their combined assessment provides a comprehensive evaluation of the optical stability of direct composite materials relevant to clinical outcomes [8–10]. Whitening mouthrinses, which have become popular in daily use, typically contain low concentrations of hydrogen peroxide, sodium hexametaphosphate, activated charcoal, or other non-peroxide agents, which are delivered to the tooth surface with the aim of preventing or reducing discoloration [11–13]. Hydrogen peroxide–based mouthrinses exert their effects primarily through oxidative mechanisms, whereas non-peroxide formulations rely on chemical chelation or surface stain removal. Despite these fundamental mechanistic differences, comparative data on their preventive effects on the optical stability of resin composites under long-term staining conditions remain limited. However, the long-term effects of these products on the color difference (Δ*E*_00_), translucency parameter (TP), and whiteness index (WID) of resin composites have not yet been fully elucidated [14, 15].

Although several studies in the literature have investigated the effects of whitening mouthrinses on the color stability of composites, most have been short-term or limited to a single material type [16, 17]. Furthermore, there is a scarcity of research that models extended and accelerated daily-use in vitro simulations involving both coffee-induced staining and whitening agents. In the present study, a three-month daily-use protocol was adopted as an extended and accelerated in vitro simulation to model repeated exposure to whitening mouthrinses. Previous studies have generally focused on the bleaching of composite materials after discoloration. However, this approach does not reflect the potential protective effects of whitening mouthrinses during daily and long-term use. Therefore, the present study aims to evaluate the preventive efficacy of whitening mouthrinses against coffee-induced discoloration and their influence on optical stability in different types of resin composite materials, based on key optical parameters.

In line with this, the null hypotheses tested in the present study are as follows:

- H₀₁: The use of whitening mouthrinses does not cause a statistically significant change in the color difference (Δ*E*_00_) values of composite resins exposed to coffee.
- H₀₂: The use of whitening mouthrinses does not significantly alter the translucency parameter (TP) values of composite resins.
- H₀₃: The use of whitening mouthrinses does not cause a statistically significant change in the whiteness index (ΔWID) of composite resins.

## Materials and methods

### Study design and sample size

The compositions, classifications, and principal characteristics of the tested composites and whitening mouthrinses are summarized in Table 1. The sample size was calculated using G*Power 3.1.9.2 software based on an effect size (*f*) of 0.3871 and a power value of 0.80. At a 95% confidence level, a minimum of 13 specimens per subgroup was estimated (*n* = 195).

**Table 1:** Composition and type of the tested materials and solutions

Product	Manufacturer/origin	Type/classification	Principal composition and characteristics
Filtek One Bulk Fill (FOB)	3 M Oral Care, USA	Bulk-fill nanohybrid composite	Monomer matrix: AUDMA, AFM, UDMA, Bis-EMA; filler: silica/zirconia cluster (~ 82 wt%, 68 vol%), mean particle size ≈ 0.6 µm. Designed for deep-cure bulk-fill applications with optimized polymerization stress and opacity control
Charisma Diamond One (CDO)	Kulzer GmbH, Germany	Universal one-shade microhybrid composite	Matrix: TCD-DI-HEA, Bis-GMA, TEGDMA, UDMA; inorganic glass and silica fillers (~ 81 wt%). Low-yellowing monomer system enhances shade adaptation and color stability
Estellite Sigma Quick (ESQ)	Tokuyama Dental, Japan	Nanohybrid composite (RAP technology)	Matrix: Bis-GMA, TEGDMA; spherical silica-zirconia fillers (82 wt%, 71 vol%, ≈ 200 nm). Uses Radical Amplified Photopolymerization (RAP) initiator system for high conversion and optical homogeneity
Listerine Advanced White (LAW)	Johnson & Johnson, Skillman, NJ, USA	Hydrogen-peroxide–based whitening mouthrinse	Hydrogen peroxide (~ 1.5%), sodium hexametaphosphate, sorbitol, flavoring agents, poloxamer 407, sodium fluoride, menthol, eucalyptol, thymol, and water. Acts via oxidative cleavage of chromogenic molecules
Colgate Optic White (COW)	Colgate-Palmolive, Switzerland	Hydrogen-peroxide–based whitening mouthrinse	Hydrogen peroxide (~ 2.0%), sodium fluoride (0.05%), phosphates, polyethylene glycol, sorbitol, flavoring agents, water. Provides mild bleaching through oxidative and chelating mechanisms
R.O.C.S. Whitening Mouthrinse (ROC)	WDS Laboratories, Monaco	Non-peroxide enzymatic whitening rinse	Calcium glycerophosphate, xylitol, bromelain enzymes, lactoperoxidase complex, essential oils, and water. Provides mechanical surface-cleaning rather than oxidative bleaching

Sample size calculation was performed using GPower software (version 3.1.9.2; Heinrich Heine University Düsseldorf, Germany). The effect size (Cohen’s *f* = 0.3871) was determined based on previously published in-vitro studies investigating color stability and optical changes of resin composites under staining and whitening protocols, in which similar experimental designs and outcome measures were used. In these studies, effect sizes were either directly reported or derived from group differences and variability parameters and subsequently applied in a priori power analyses using Gpower [18, 19]. A statistical power of 80% and an alpha level of 0.05 were adopted, consistent with recent in-vitro restorative materials research.

### Specimen preparation

Three different resin composite materials were used:

- Charisma Diamond One―CDO (Kulzer, Germany)
- Estellite Sigma Quick A2―ESQ (Tokuyama, Japan)
- Filtek One Bulk Fill A2―FOB (3 M Oral Care, USA)

A total of 195 disc-shaped specimens were prepared, with 65 samples from each material (*n* = 13 per subgroup). For each composite material, the specimens were randomly allocated into five subgroups according to the immersion protocol: distilled water (negative control), coffee (positive control), coffee followed by R.O.C.S., coffee followed by Colgate Optic White (COW), and coffee followed by Listerine Advanced White (LAW). The specimens were fabricated using silicone molds with a diameter of 8 mm and a thickness of 2 mm. Each composite material was placed between two microscope glass slides and compressed with a transparent polyester strip, then polymerized from both sides using the same LED curing unit (D-Light Pro, GC, Tokyo, Japan) at a constant light intensity of 1400 mW/cm^2^; however, the exposure time was selected according to the manufacturer’s recommendations for each respective composite material. To prevent the formation of an oxygen inhibition layer, the specimens were prepared using the double-glass slide technique, applying pressure so that a transparent polyester strip covered the surface of each sample. After polymerization, both surfaces of the specimens were polished in accordance with the manufacturer’s instructions using aluminum oxide–coated polishing discs (OptiDisc, KerrHawe, Bioggio, Switzerland). The specimens were polished under water cooling at a low rotational speed (5000 rpm) with light, passive pressure. The final thickness of each specimen was verified using a digital caliper, and the samples were randomly divided into five subgroups. For standardization purposes, all specimen preparations were performed by an experienced dentist.

### Color measurement

Initial color measurements were performed after the specimens were immersed in distilled water at 37 °C for 24 h to allow post-curing and water saturation before measurement. Color assessments were conducted using a Vita Easyshade V spectrophotometer (Vita Zahnfabrik, Bad Säckingen, Germany) based on the CIE Lab* color system. Measurements were taken against black (*L* ≈ 1, *a* ≈ 0, b* ≈ 0), white (*L* ≈ 97, *a* ≈ 0, b* ≈ 0), and neutral gray (*L* ≈ 50, *a* ≈ 0, b* ≈ 0)** backgrounds.

These background values were selected in accordance with CIE and ISO 11664-4 standards to maximize measurement contrast and ensure the accuracy of translucency parameter (TP) calculations [9, 10]. Each specimen was measured three times on each background, and the mean values were recorded. The spectrophotometer was calibrated before each measurement according to the manufacturer’s instructions.

### Experimental procedure

The staining model was conducted in a daily coffee/whitening mouthrinse cycle for a duration of three months. The coffee solution was prepared by dissolving the contents of a commercially available single-use coffee sachet (3.6 g, as specified by the manufacturer; Nescafé Classic, Nestlé, Türkiye) in 300 mL of drinking water at a temperature of 60 ± 2 °C, which was verified using a digital thermometer with an immersion probe (Testo 108, Germany). The specimens in the positive control group, which were stored in distilled water, were immersed in the coffee solution for 48 min daily, representing a cumulative exposure duration derived from typical daily coffee consumption patterns reported in the literature, based on an estimated average of 3–4 coffee intake events per day with an approximate contact time of 10–15 min per serving, with gentle manual agitation performed by gently swirling the containers every 5 min during immersion. At the end of each cycle, the samples were rinsed under running distilled water for approximately 10 s in a standardized manner and then returned to their respective distilled water containers.

Similarly, in the whitening mouthrinse groups, the specimens—also stored in distilled water—were immersed in coffee for 48 min following the same cumulative daily exposure rationale, then rinsed with distilled water, and subsequently subjected to gentle manual agitation for 60 s, in accordance with the manufacturer’s recommended mouthrinse usage instructions..

- R.O.C.S. Whitening Mouthrinse (R.O.C.S., WDS Lab, Monaco)
- Colgate Optic White Mouthwash (COW) (Colgate-Palmolive Co., New York, USA)
- Listerine Advanced White Mouthrinse (LAW) (Johnson & Johnson, New Brunswick, USA)

The specimens in the negative control group were stored only in distilled water. All solutions were renewed daily, and color measurements (L*, a*, and b*) were repeated at the end of the 1st, 2nd, and 3rd months, following the same measurement protocol.

According to the manufacturers’ official ingredient disclosures, Listerine Advanced White and Colgate Optic White contain low concentrations of hydrogen peroxide (approximately 1.5% and 2.0%, respectively), whereas R.O.C.S. Whitening Mouthrinse is a non-peroxide formulation. The classification of the tested mouthrinses as peroxide-based or non-peroxide-based was therefore made strictly based on manufacturer-reported compositions (Table 1**)**.

### Calculation of color difference, translucency, and whiteness index

Color, translucency, and whiteness parameters were calculated using the following formulas:

(a) Color difference (Δ*E*_00_): according to the CIE DE 2000 standard:

$$\Delta E_{00} = \sqrt {\left( {\frac{{\Delta L^{\prime}}}{{k_{{\mathrm{L}}} S_{{\mathrm{L}}} }}} \right)^{2} + \left( {\frac{{\Delta C^{\prime}}}{{k_{{\mathrm{C}}} S_{{\mathrm{C}}} }}} \right)^{2} + \left( {\frac{{\Delta H^{\prime}}}{{k_{{\mathrm{H}}} S_{{\mathrm{H}}} }}} \right)^{2} + R_{T} \left( {\frac{{\Delta C^{\prime}}}{{k_{{\mathrm{C}}} S_{{\mathrm{C}}} }}} \right)\left( {\frac{{\Delta H^{\prime}}}{{k_{{\mathrm{H}}} S_{{\mathrm{H}}} }}} \right)}$$

Here, ΔL′, ΔC′, and ΔH′ represent the differences in lightness, chroma, and hue, respectively. In all calculations, the parametric factors were set as *k*_L_ = *k*_C_ = *k*_H_ = 1, in accordance with the CIEDE2000 standard and the commonly accepted methodology in dental literature [9, 19].

## (b) Translucency parameter (TP)

Calculated using the measurements obtained over black and white backgrounds:

$${\mathrm{TP}} = \sqrt {(L_{{\mathrm{B}}}^{*} - L_{{\mathrm{W}}}^{*} )^{2} + (a_{{\mathrm{B}}}^{*} - a_{{\mathrm{W}}}^{*} )^{2} + (b_{{\mathrm{B}}}^{*} - b_{{\mathrm{W}}}^{*} )^{2} }$$

Here, the subscript B refers to the measurement on the black background, and W refers to the measurement on the white background. A higher TP value indicates greater light transmittance of the material.

## (c) Whiteness index (WI)

According to the CIE formula, the whiteness index is calculated as:

$${\mathrm{WID}} = 0.511L{*} - 2.324a{*} - 1.100b{*}$$

The change in whiteness over time is expressed as:

$$\Delta {\mathrm{WI}} = {\mathrm{WI}}_{t} - {\mathrm{WI}}_{0}$$

### Statistical analysis

Data were analyzed using IBM SPSS Statistics 25 software. Normality and homogeneity of variance were assessed using the Shapiro–Wilk and Levene tests, respectively. Parametric data were analyzed using one-way ANOVA or mixed-design repeated-measures ANOVA, while non-parametric data were evaluated using the Kruskal–Wallis and Friedman tests. Post-hoc comparisons were performed with the Bonferroni correction.

Effect sizes were calculated (Kendall’s W for Friedman tests and Cohen’s *d* for comparisons relative to the distilled water control), and 95% confidence intervals were reported for 3-month outcomes. Associations among Δ*E*_00_, ΔTP, and ΔWID values at 3 months were analyzed using Spearman’s rank correlation. Statistical significance was set at *p* < 0.05.

## Results

### Color difference (Δ***E***_00_) results

The effects of time (baseline, 1st, 2nd, and 3rd months) and solution on Δ*E*_00_ values were evaluated using a mixed-design repeated-measures ANOVA, in which time was considered the within-subject factor and solution was treated as the between-subject factor. All calculations were performed using the CIE DE 2000 formula. For each specimen, Δ*E*_00_ values were calculated for the 1st, 2nd, and 3rd months, taking the initial measurement as the reference point. Color difference (Δ*E*_00_) and whiteness index difference (ΔWID) analyses were conducted using measurements obtained on the neutral gray background, whereas translucency parameter (ΔTP) values were calculated based on measurements performed on black and white backgrounds, in accordance with CIE and ISO recommendations. Three composite materials (FOB, CDO, and ESQ) and five immersion protocols (coffee staining, distilled water storage, and coffee followed by R.O.C.S., COW, or LAW mouthrinse exposure) were evaluated.

**Fig. 1:**
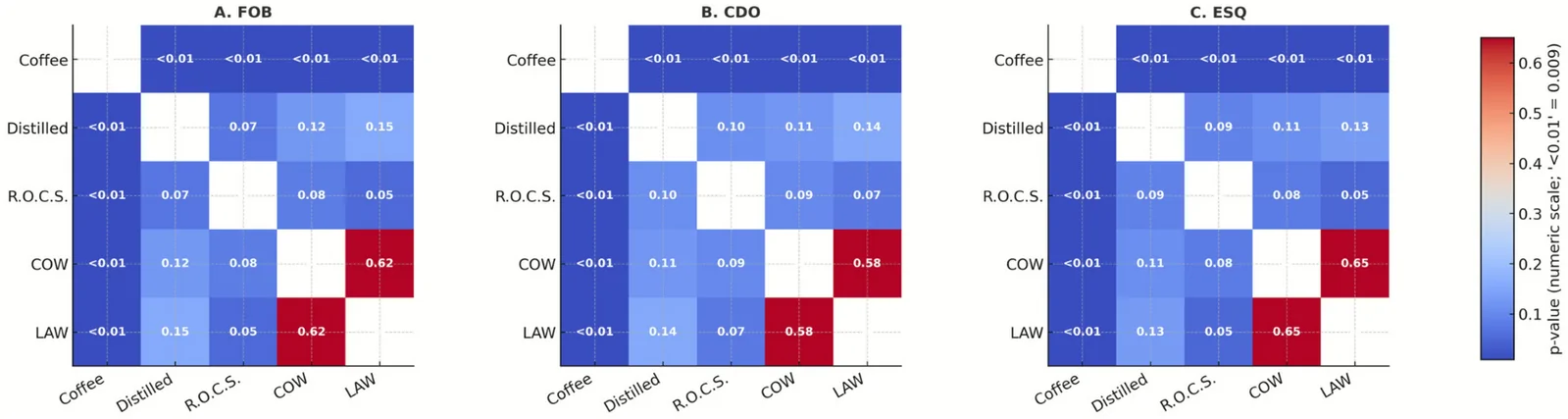
Post-hoc significance heatmaps (Δ*E*_00_, 3rd month) for composite resins

According to the Kruskal–Wallis test, statistically significant differences were found among all materials in terms of Δ*E*_00_ values at the 3rd month (*p* < 0.05). Significant differences among the solutions were observed within the FOB (*p* < 0.001), CDO (*p* < 0.01), and ESQ (*p* < 0.01) groups (Table 2). Post-hoc analyses revealed that the Coffee group consistently exhibited the highest Δ*E*_00_ values across all composite materials, indicating pronounced discoloration. In contrast, the whitening mouthrinse groups, particularly LAW and COW, showed significantly lower Δ*E*_00_ values compared with the Coffee group (*p* < 0.01), with no statistically significant difference between LAW and COW (*p* > 0.05). The R.O.C.S. group demonstrated intermediate Δ*E*_00_ values, which were higher than those of LAW but lower than the Coffee group (Fig. 1). The Δ*E*_00_ values showed a gradual increase from the 1st to the 3rd month, with the most pronounced rise observed in the Coffee group. Among the materials, ESQ showed the lowest mean Δ*E*_00_ values across all solutions. Based on the gray background measurements, LAW consistently showed the lowest Δ*E*_00_ values across all materials. Overall, ESQ showed lower mean Δ*E*_00_ values than FOB and CDO across all solutions (*p* < 0.05). According to previously established clinical acceptability thresholds, Δ*E*_00_ values above 1.8 are considered clinically unacceptable for direct restorative materials. In the present study, the Coffee groups exceeded this acceptability threshold, whereas the LAW and COW groups exhibited Δ*E*_00_ values close to or slightly above this limit at the 3-month evaluation [9, 19]. The magnitude of time-related color changes was further supported by Kendall’s W effect size values and corresponding 95% confidence intervals (Table 2**)**.

**Table 2:** Mean color changes (Δ*E*_00_) at 1, 2, and 3 months

Material	Solution	1st Month (mean ± SD)	2nd Month (mean ± SD)	3rd Month (mean ± SD)	*p*-Value^†^	Kendall’s W	95% CI
FOB	Distilled water	0.90 ± 0.31	1.08 ± 0.39	1.33 ± 0.29	0.21 (n.s.)	0.18	0.05–0.33
	Coffee	2.01 ± 0.59	2.41 ± 0.71	2.79 ± 0.77	< 0.001	0.63	0.49–0.75
	R.O.C.S	1.69 ± 0.52	2.18 ± 0.61	2.77 ± 0.69	0.02	0.52	0.37–0.66
	COW	1.49 ± 0.41	1.98 ± 0.50	2.42 ± 0.57	0.03	0.47	0.32–0.61
	LAW	1.31 ± 0.38	1.78 ± 0.47	2.09 ± 0.48	0.04	0.45	0.30–0.59
CDO	Distilled water	0.98 ± 0.33	1.19 ± 0.41	1.41 ± 0.32	0.24 (n.s.)	0.20	0.07–0.35
	Coffee	2.32 ± 0.61	2.68 ± 0.74	3.01 ± 0.81	< 0.001	0.66	0.52–0.77
	R.O.C.S	1.82 ± 0.53	2.39 ± 0.59	2.88 ± 0.63	0.01	0.55	0.40–0.69
	COW	1.61 ± 0.42	2.18 ± 0.52	2.53 ± 0.58	0.03	0.49	0.34–0.63
	LAW	1.39 ± 0.40	1.88 ± 0.48	2.28 ± 0.51	0.04	0.46	0.31–0.60
ESQ	Distilled water	0.82 ± 0.29	1.07 ± 0.36	1.29 ± 0.27	0.19 (n.s.)	0.17	0.04–0.31
	Coffee	2.03 ± 0.54	2.47 ± 0.68	2.92 ± 0.79	< 0.001	0.61	0.46–0.73
	R.O.C.S	1.63 ± 0.50	2.21 ± 0.57	2.69 ± 0.61	0.02	0.51	0.36–0.65
	COW	1.41 ± 0.43	1.99 ± 0.48	2.31 ± 0.49	0.03	0.48	0.33–0.62
	LAW	1.22 ± 0.39	1.71 ± 0.41	2.01 ± 0.46	0.04	0.44	0.29–0.58

^†^Time-related changes in Δ*E*_00_ values were analyzed using the Friedman test

Kendall’s W was reported as the effect size, and 95% confidence intervals (CI) were calculated using bootstrap resampling. n.s.: not significant

### Translucency difference (ΔTP) results

In this section, the translucency difference (ΔTP) values of three composite materials (FOB, CDO, ESQ) were evaluated after exposure to five different solutions (Coffee, R.O.C.S., COW, LAW, and Distilled water). The Coffee group showed the most negative ΔTP values across all materials. In contrast, the whitening agents (R.O.C.S., COW, and LAW) maintained translucency with no statistically significant changes in ΔTP values (*p* > 0.05). The Distilled water group produced results similar to those of the whitening groups. The general trend observed was: ΔTP (Coffee) < ΔTP (R.O.C.S. ≈ COW ≈ LAW ≈ Distilled water) **(**Table 3**).**

**Table 3:** Changes in translucency parameter (TP) and ΔTP values of composite resins over time

Material	Solution	TP₀ (mean ± SD)	ΔTP₁ (mean ± SD)	ΔTP_2_ (mean ± SD)	ΔTP_3_ (mean ± SD)	95% CI	Effect size vs DW (*d*)	Difference (3rd month)
FOB	Coffee	15.8 ± 0.4	− 1.50 ± 0.25	− 1.80 ± 0.27	− 2.08 ± 0.35	− 2.30 to − 1.86	2.3 (very large)	a
	R.O.C.S	15.7 ± 0.5	− 0.40 ± 0.15	− 0.60 ± 0.18	− 0.83 ± 0.18	− 0.94 to − 0.72	0.9 (large)	b
	COW	15.32 ± 0.2	− 0.30 ± 0.10	− 0.50 ± 0.15	− 0.68 ± 0.16	− 0.78 to − 0.58	0.7 (moderate)	b
	LAW	15.3 ± 0.75	− 0.20 ± 0.12	− 0.40 ± 0.14	− 0.62 ± 0.15	− 0.71 to − 0.53	0.6 (moderate)	b
	Distilled W	15.8 ± 0.4	− 0.10 ± 0.08	− 0.20 ± 0.10	− 0.28 ± 0.11	− 0.35 to − 0.21	Reference	b
*p* (Kruskal–Wallis) < 0.01—The Coffee group differed significantly from all others								
Material	Solution	TP₀ (mean ± SD)	ΔTP₁ (mean ± SD)	ΔTP_2_ (mean ± SD)	ΔTP_3_ (mean ± SD)	95% CI	Effect size vs DW (*d*)	Difference (3rd month)
CDO	Coffee	16.2 ± 0.5	− 1.10 ± 0.20	− 1.50 ± 0.22	− 1.86 ± 0.31	− 2.05 to − 1.67	6.35 (very large)	a
	R.O.C.S	16.1 ± 0.5	− 0.30 ± 0.14	− 0.60 ± 0.16	− 0.71 ± 0.20	− 0.83 to − 0.59	2.35 (very large)	b
	COW	16.0 ± 0.5	− 0.30 ± 0.12	− 0.60 ± 0.15	− 0.70 ± 0.17	− 0.80 to − 0.60	2.62 (very large)	b
	LAW	16.1 ± 0.5	− 0.20 ± 0.10	− 0.40 ± 0.13	− 0.52 ± 0.18	− 0.63 to − 0.41	1.37 (large)	b
	Distilled W	16.2 ± 0.4	− 0.10 ± 0.08	− 0.20 ± 0.09	− 0.31 ± 0.12	− 0.38 to − 0.23	Reference	b
*p* (Kruskal–Wallis) = 0.02—Coffee significantly reduced translucency								
Material	Solution	TP₀ (mean ± SD)	ΔTP₁ (mean ± SD)	ΔTP_2_ (mean ± SD)	ΔTP_3_ (mean ± SD)	95% CI	Effect size vs DW (*d*)	Difference (3rd month)
ESQ	Coffee	17.0 ± 0.5	− 1.20 ± 0.22	− 1.60 ± 0.25	− 2.00 ± 0.28	− 2.17 to − 1.83	− 7.76 (very large)	a
	R.O.C.S	16.9 ± 0.5	− 0.40 ± 0.13	− 0.60 ± 0.15	− 0.78 ± 0.22	− 0.91 to − 0.64	− 2.71 (very large)	b
	COW	16.8 ± 0.5	− 0.30 ± 0.12	− 0.50 ± 0.14	− 0.60 ± 0.15	− 0.69 to − 0.51	− 2.25 (very large)	b
	LAW	16.9 ± 0.5	− 0.20 ± 0.10	− 0.30 ± 0.12	− 0.42 ± 0.19	− 0.53 to − 0.30	− 0.75 (moderate–large)	b
	Distilled W	17.0 ± 0.5	− 0.10 ± 0.09	− 0.20 ± 0.10	− 0.30 ± 0.10	− 0.36 to − 0.24	Reference	b
*p* (Kruskal–Wallis) < 0.01—Translucency loss was significant in the Coffee group								

Different superscript letters (a, b) indicate statistically significant differences among solutions within the same composite material at the 3rd month (Kruskal–Wallis test with post-hoc comparisons, *p* < 0.05)

Effect sizes are reported as absolute values (|d|) relative to distilled water and interpreted as: small (~ 0.2), moderate (~ 0.5), large (~ 0.8), very large (≥ 1.2). 95% confidence intervals (CI) refer to the mean ΔTP₃ values and were calculated using the *t*-distribution

**Fig. 2:**
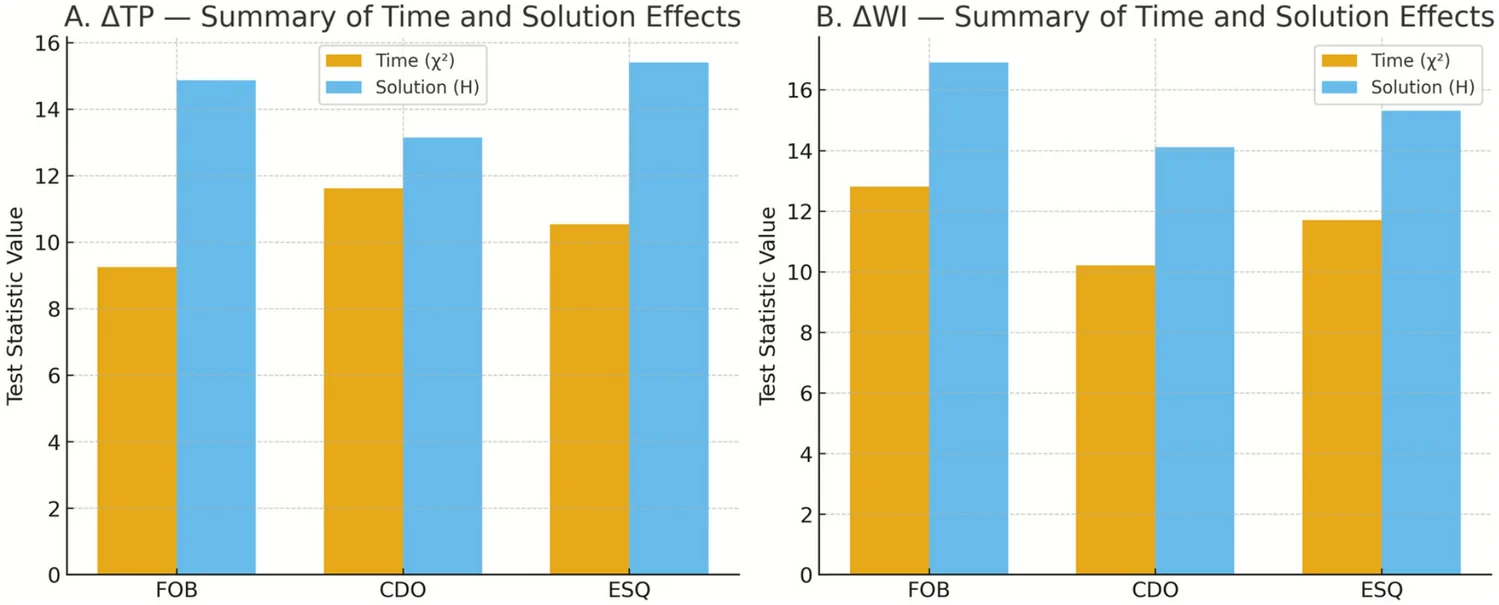
Comparative summary of time (*χ*^2^) and solution (*H*) effects on optical parameters

Bar charts summarizing the test statistic magnitudes for the effects of time (*χ*^2^) and solution (*H*) for each composite resin. Panel A illustrates the ΔTP results, while Panel B presents the ΔWID outcomes. Figure 2 presents the test statistics for the effects of time (*χ*^2^) and solution (*H*) on ΔTP and ΔWID. In both analyses, the solution factor yielded higher test statistics than the time factor (Fig. 2).

A notable decrease in ΔTP values was observed over time across all materials. In contrast, applications involving whitening solutions (R.O.C.S., COW, LAW) largely preserved translucency values, and ΔTP values in the whitening groups remained below 1. The Distilled water group showed ΔTP values comparable to those of the whitening groups. Among the tested composites, ESQ exhibited the highest initial TP value. Effect sizes and 95% confidence intervals were calculated for the 3-month values to reflect cumulative optical changes among the groups.

### Whiteness Index (WI) results

For the three composite materials (FOB, CDO, ESQ) and five immersion protocols (coffee staining, distilled water storage, and coffee followed by R.O.C.S., COW, or LAW mouthrinse exposure), the initial, 1st-, 2nd-, and 3rd-month values were evaluated. The effect of time was analyzed using the Friedman test, while the effect of solution was assessed with the Kruskal–Wallis test (Table 4). In the Coffee groups, ΔWID values decreased significantly over time (*p* < 0.05). In contrast, the whitening solutions (LAW, COW, R.O.C.S.) increased whiteness values. The Coffee solutions produced negative ΔWID values (loss of whiteness) across all materials, whereas the whitening solutions showed positive ΔWID values (increase in whiteness).

**Table 4:** Changes in whiteness index (ΔWID) of composite resins over time

Materials	Solutions	WI₀ (mean ± SD)	ΔWI₁ (mean ± SD)	ΔWI₂ (mean ± SD)	ΔWI₃ (mean ± SD)	Effect of time (*p*)	95% CI (mean)	Effect size vs DW (*d*)
FOB	Coffee	26.70 ± 0.39	− 1.5 ± 0.4	− 2.1 ± 0.5	− 3.00 ± 0.18	< 0.001	− 3.10 to − 2.89	22.47 (very large)
	R.O.C.S	27.35 ± 0.54	+ 0.8 ± 0.3	+ 1.2 ± 0.4	+ 1.47 ± 0.32	0.03	+ 1.28 to + 1.66	4.96 (very large)
	COW	27.56 ± 0.40	+ 0.7 ± 0.3	+ 1.0 ± 0.3	+ 1.38 ± 0.30	0.04	+ 1.20 to + 1.56	4.76 (very large)
	LAW	27.28 ± 0.74	+ 0.5 ± 0.2	+ 0.9 ± 0.3	+ 1.28 ± 0.24	0.05	+ 1.13 to + 1.42	5.23 (very large)
	Distilled W	27.72 ± 1.65	+ 0.1 ± 0.1	+ 0.2 ± 0.1	+ 0.30 ± 0.10	n.s	+ 0.24 to + 0.37	Reference
CDO	Coffee	36.92 ± 1.00	− 1.2 ± 0.4	− 1.8 ± 0.5	− 2.22 ± 0.58	0.002	− 2.57 to − 1.87	5.86 (very large)
	R.O.C.S	34.36 ± 0.47	+ 0.6 ± 0.2	+ 0.9 ± 0.3	+ 1.19 ± 0.31	0.04	+ 1.00 to + 1.38	4.38 (very large)
	COW	36.84 ± 0.66	+ 0.5 ± 0.2	+ 0.8 ± 0.3	+ 1.08 ± 0.27	0.04	+ 0.92 to + 1.25	4.49 (very large)
	LAW	37.32 ± 0.63	+ 0.4 ± 0.2	+ 0.8 ± 0.2	+ 1.00 ± 0.25	0.05	+ 0.85 to + 1.15	4.36 (very large)
	Distilled W	36.21 ± 1.1	+ 0.0 ± 0.1	+ 0.1 ± 0.1	+ 0.20 ± 0.08	n.s	+ 0.15 to + 0.25	Reference
ESQ	Coffee	20.15 ± 0.40	− 1.4 ± 0.3	− 2.0 ± 0.5	− 2.47 ± 0.50	< 0.001	− 2.77 to − 2.17	7.97 (very large)
	R.O.C.S	20.48 ± 0.97	+ 0.9 ± 0.3	+ 1.3 ± 0.4	+ 1.58 ± 0.39	0.03	+ 1.35 to + 1.81	4.25 (very large)
	COW	20.26 ± 0.21	+ 0.8 ± 0.3	+ 1.2 ± 0.3	+ 1.49 ± 0.30	0.04	+ 1.31 to + 1.67	5.00 (very large)
	LAW	19.77 ± 0.31	+ 0.6 ± 0.2	+ 1.0 ± 0.3	+ 1.28 ± 0.27	0.05	+ 1.11 to + 1.44	4.41 (very large)
	Distilled W	18.31 ± 0.15	+ 0.2 ± 0.1	+ 0.3 ± 0.1	+ 0.39 ± 0.08	n.s	+ 0.35 to + 0.44	Reference

Positive ΔWID values indicate an increase in whiteness, whereas negative values indicate a loss of whiteness. Effect of time was assessed using the Friedman test. 95% confidence intervals (CI) refer to the mean ΔWI₃ values and were calculated using the *t*-distribution (*n* = 13). Effect sizes are expressed as Cohen’s *d* (absolute values) relative to distilled water and interpreted as small (~ 0.2), moderate (~ 0.5), large (~ 0.8), and very large (≥ 1.2). n.s.: not significant

The Coffee solution significantly reduced the whiteness index (WID) across all materials, and this effect was found to be statistically significant (ΔWID negative, *p* < 0.05). In contrast, the whitening agents—LAW, COW, and R.O.C.S.—caused a notable increase in WID values, resulting in positive ΔWID values (*p* < 0.05). The Distilled water group, serving as the control, showed no significant change over time and remained stable throughout the study period (*p* > 0.05). Although an increase in ΔWID was observed in all whitening groups, the mean values at the 3-month interval ranged between + 1.0 and + 1.6. Across all composites, the whitening groups showed positive ΔWID values, while the Coffee groups showed negative ΔWID values.

**Fig. 3:**
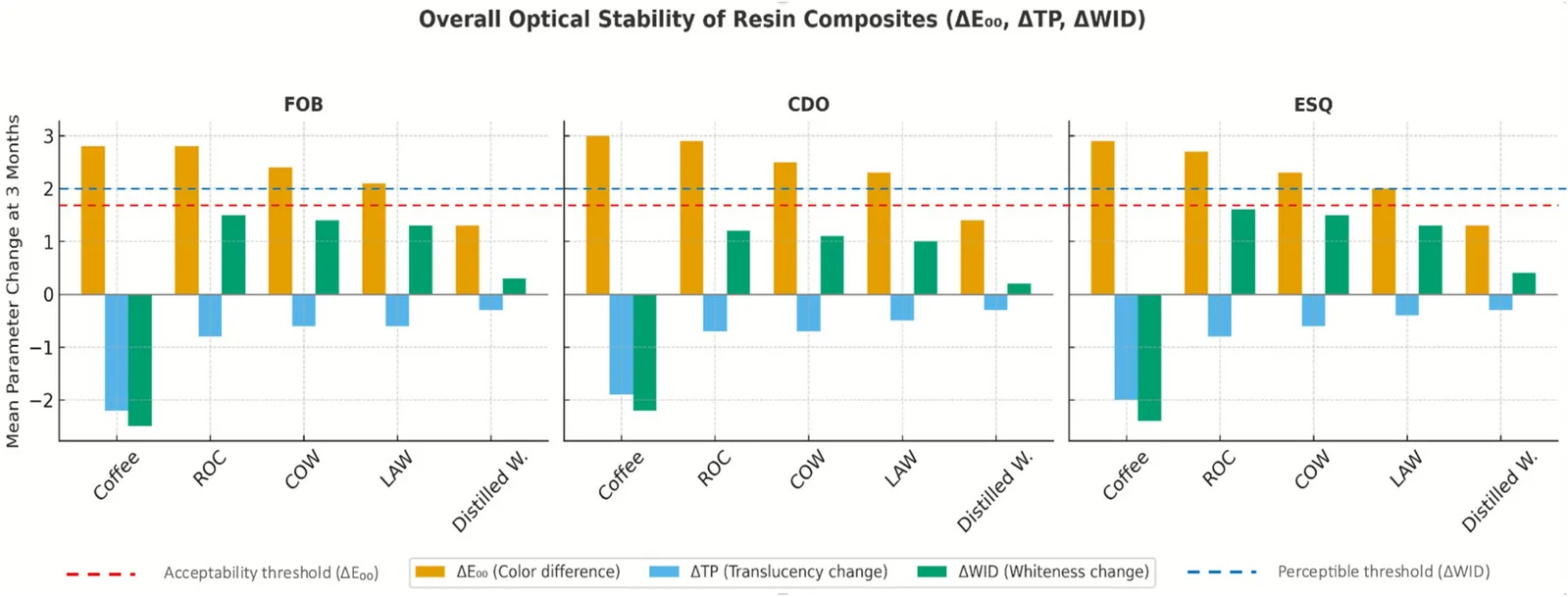
Overall optical stability of resin composites (Δ*E*_00_, ΔTP, and ΔWID)

Among the materials, ESQ showed higher positive ΔWID values than CDO and FOB. LAW showed lower Δ*E*_00_ values and positive ΔWID values across all materials. A comparative summary of the optical parameter changes (Δ*E*_00_, ΔTP, and ΔWID) for all materials and solutions is presented in Fig. 3**.**

### Correlation analysis of optical parameters

Spearman correlation analysis showed a moderate negative correlation between color change (Δ*E*_00₃_) and translucency change (ΔTP₃) for all composite materials (ρ =  − 0.55 to − 0.59, *p* < 0.001). A weak but significant positive correlation was observed between ΔTP₃ and ΔWID₃ (ρ = 0.28–0.32, *p* < 0.05). No significant correlation was found between Δ*E*_00₃_ and ΔWID₃ (*p* > 0.05) (Table 5).

**Table 5:** Spearman correlation analysis among color change (Δ*E*_00_), translucency change (ΔTP), and Whiteness index change (ΔWID) at 3 months

Material	Variables	Spearman ρ	*p*-Value
Filtek	Δ*E*_00₃_ vs ΔTP₃	− 0.58	< 0.001
	ΔTP₃ vs ΔWI₃	0.28	0.026
	Δ*E*_00₃_ vs ΔWI₃	0.07	0.58
Charisma	Δ*E*_00₃_ vs ΔTP₃	− 0.55	< 0.001
	ΔTP₃ vs ΔWI₃	0.32	0.010
	Δ*E*_00₃_ vs ΔWI₃	0.10	0.44
Estellite	Δ*E*_00₃_ vs ΔTP₃	− 0.59	< 0.001
	ΔTP₃ vs ΔWI₃	0.29	0.018
	Δ*E*_00₃_ vs ΔWI₃	− 0.05	0.70

Correlation strength was interpreted as weak (|ρ|< 0.30), moderate (0.30 ≤|ρ|< 0.60), or strong (|ρ|≥ 0.60)

## Discussion

This study evaluated the protective effects of whitening mouthrinses against coffee-induced discoloration in different resin composites, using the parameters Δ*E*_00_, ΔTP, and ΔWID. The findings revealed that the whitening solutions produced statistically significant effects on color difference (Δ*E*_00_) and whiteness index (ΔWID), but did not cause a significant change in translucency difference (ΔTP). Accordingly, the H₀₁ and H₀₃ hypotheses were rejected, while H₀₂ was accepted. Although the differences in Δ*E*_00_ and ΔWID were statistically significant, their magnitudes remained close to or below the clinically perceptible thresholds, indicating that the observed optical changes were limited in clinical relevance. These results suggest that whitening mouthrinses positively influence color stability and whiteness, whereas their effect on light transmittance is neither statistically nor clinically significant.

The obtained Δ*E*_00_ results demonstrated that the Coffee groups exhibited a pronounced discoloration across all materials, whereas the whitening solutions significantly reduced this effect. In particular, the LAW group showed the lowest Δ*E*_00_ values, indicating the best color stability. This finding is consistent with previous studies reporting that hydrogen peroxide–containing mouthrinses contribute to the maintenance of color stability [5, 11]. The oxidative agents present in whitening solutions are thought to dissolve organic pigments accumulated on the composite surface, thereby reducing discoloration. Similarly, previous studies have confirmed the effectiveness of hydrogen peroxide–containing mouthrinses in maintaining color stability, whereas non-peroxide formulations were reported to provide only limited whitening [20, 21]. To enhance interpretability, effect sizes and 95% confidence intervals were reported alongside *p*-values, allowing the magnitude and precision of the observed changes to be evaluated beyond statistical significance alone. This is particularly relevant for optical outcomes where statistically significant differences may still remain below perceptibility/acceptability thresholds.

The duration of exposure represents an important methodological consideration in in vitro mouthrinse studies. Previous investigations have predominantly relied on short-term immersion protocols ranging from 24 h to one week, which are often interpreted as accelerated equivalents of long-term clinical use. In contrast, the present study employed a three-month daily cyclic exposure model to provide an extended observation period under repeated-use conditions. This protocol was selected to better simulate cumulative exposure to whitening mouthrinses over time, while remaining feasible within an in vitro experimental design. Although not intended to replicate true long-term clinical use, the adopted model exceeds the duration of most previously reported in vitro studies and allows for a more comprehensive evaluation of optical stability changes. The differences between the present extended daily-use cyclic in vitro model and short-term immersion studies can be attributed to cumulative pigment diffusion and surface–matrix interactions in resin composites. Short-term protocols mainly reflect early surface adsorption and may therefore overestimate the immediate whitening efficacy of peroxide-containing solutions. In contrast, repeated daily exposure allows deeper pigment penetration into the resin matrix, resulting in more moderate and clinically realistic changes in color difference and whiteness parameters.

The higher Δ*E*_00_ values observed for the R.O.C.S. solution compared to the other whitening groups can be attributed to its formulation, which contains non-peroxide components (e.g., calcium glycerophosphate, xylitol, etc.) instead of hydrogen peroxide. Although these agents provide a mechanical surface-cleaning effect, their oxidative whitening capability is weak, resulting in a limited potential to reduce color differences. This trend is consistent with previous reports indicating that hydrogen peroxide–based treatments reduce color differences in hybrid composites, although the experimental conditions and application protocols differ from those used in the present study [4]. Previous studies have indicated that the effectiveness of non-peroxide products is limited to superficial mechanical stain removal, and that such formulations are insufficient to prevent the deeper diffusion of organic pigments into the resin matrix [21, 22].

From a clinical perspective, Δ*E*_00_ threshold values reported by Paravina et al. indicate that the 50:50% perceptibility threshold is approximately 0.8, while the clinical acceptability threshold is around 1.8. Similar acceptability limits have also been reported by Khashayar et al. In the present study, the Δ*E*_00_ values observed in the LAW and COW groups remained close to the acceptability threshold at the end of the third month, suggesting borderline but clinically acceptable color stability for composite restorations [9, 19]. The magnitude of time-dependent discoloration was further supported by the Kendall’s W effect size estimates with corresponding 95% confidence intervals, indicating a cumulative staining effect over the 3-month protocol.

In the present model, pigment accumulation in coffee-exposed composites may have disrupted optical reflection pathways, thereby contributing to the observed reduction in the translucency parameter. However, Fidan et al. reported that the effect of whitening mouthrinses on translucency varies depending on the type of material: while no significant difference was observed in the LAW group, an increase in translucency values was noted for the hydrogen peroxide–containing Crest 3D White group [12]. This suggests that, although whitening agents oxidize surface pigments, their influence on the deeper optical structure of the material is limited. Considering that translucency is primarily determined by the refractive index mismatch between filler particles and the resin matrix, short-term chemical interactions are not expected to induce marked alterations in this parameter [4, 6]. In addition, variations in filler size distribution have been shown to directly influence the optical transmittance of composite materials [23]. Although hydrogen peroxide has been reported to exert potential surface-degrading effects on resin-based materials, such effects are primarily limited to superficial resin matrix components when low concentrations are used. In the present study, the minimal changes observed in translucency may be explained by the high filler loading and homogeneous filler distribution of the tested composites, which likely acted as a protective barrier against oxidative penetration. As a result, possible surface alterations did not substantially affect the filler–matrix refractive index relationship or light transmittance pathways, leading to clinically negligible changes in translucency [4, 6, 22]. Reporting effect sizes and 95% confidence intervals for the 3-month ΔTP outcomes further supports that translucency changes in the mouthrinse groups were small in magnitude compared with coffee exposure **(**Table 3**)**.

The color measurement results indicated that the whitening solutions produced a pronounced whitening tendency on the surfaces of the composite materials. In the Coffee group, all materials exhibited a decrease in whiteness index (negative change), whereas the LAW, COW, and R.O.C.S. groups demonstrated an increase in whiteness index (positive ΔWID). This trend is consistent with the findings of Pérez et al., who reported a clinically perceptible whiteness difference threshold (WID ≈ 2.6) [10]. In addition, previous studies have confirmed the color-lightening effect of hydrogen peroxide–based mouthrinses [17].

It has been observed that whitening mouthrinses accelerate the oxidation of surface chromophores, resulting in a rapid increase in ΔWID values over a short period [21]. Moreover, peroxide-based formulations have been shown to sustain this whitening effect for more than two weeks, indicating greater durability compared with non-peroxide systems [18]. A ΔWID value of approximately + 2 or higher has been suggested to represent a clinically perceptible whitening effect [10]. In the present study, the ΔWID increase observed in the LAW group ranged between + 1.0 and + 1.3, which is below this clinical threshold. Therefore, this change should be interpreted as a mild whitening tendency rather than a visually perceptible effect. The 95% confidence intervals around the 3-month ΔWID values reinforce that the observed whitening tendency remained modest and below commonly cited perceptibility limits (Table 4). This effect of whitening solutions is attributed to the oxidation of organic chromophores accumulated on the surface, converting them into colorless compounds through hydrogen peroxide–mediated oxidative reactions. The correlation analysis offers additional insight into how these optical outcomes co-vary after prolonged staining and preventive rinsing. A moderate negative association between Δ*E*_00₃_ and ΔTP₃ suggests that greater discoloration tends to coincide with greater translucency loss, which is compatible with cumulative pigment uptake and increased light scattering within the resin matrix. In contrast, the weak positive correlation between ΔTP₃ and ΔWID₃ indicates that small increases in whiteness may occur alongside minor translucency shifts, although the relationship remained limited in magnitude. Notably, Δ*E*_00₃_ was not significantly correlated with ΔWID₃, supporting the interpretation that color difference and whiteness index capture partly distinct perceptual dimensions and may not change in parallel under identical exposure conditions **(**Table 5**).**

The ESQ material exhibited the lowest Δ*E*_00_ and the highest ΔTP values across all parameters, which can be attributed to its nanohybrid structure, characterized by a homogeneous filler distribution and high light transmittance. In contrast, the bulk-fill composites (FOB and CDO) showed higher Δ*E*_00_ values, likely due to their larger filler particles, which increase light scattering and consequently reduce translucency. The literature also reports that the refractive index compatibility between filler particles and the resin matrix plays a determinant role in the optical performance of composites [6, 8]. It has been shown that a homogeneous distribution of nano-sized fillers in nanohybrid systems reduces light scattering and enhances optical stability [8, 24]. This observation is consistent with the findings of the present study, particularly for the ESQ material. The findings indicate that the regular use of whitening mouthrinses may help reduce color changes caused by pigment-containing beverages such as coffee. From a clinical standpoint, Δ*E*_00_ values below 3 and a ΔWID increase of approximately + 2 or higher suggest that restorations remain within aesthetically acceptable limits. Accordingly, hydrogen peroxide–based solutions, such as LAW and COW, may be considered supportive agents for maintaining color stability in clinical applications. However, it has been reported that prolonged use of peroxide-based products with low pH may lead to micromorphological alterations on the enamel surface. Therefore, these solutions should be used in a controlled manner and for limited periods of time [21, 22].

The effect of whitening solutions is closely related to their chemical composition. Hydrogen peroxide–containing solutions, such as LAW and COW, act by oxidizing chromogenic compounds, thereby producing a lightening effect. In contrast, R.O.C.S., which is non-peroxide-based, primarily exerts a mechanical surface-cleaning effect. Coffee-induced discoloration occurs through the diffusion of pigments, such as tannins and polyphenols, into the composite matrix; therefore, the surface-limited action of whitening agents does not alter translucency, but instead leads to an increase in the whiteness index [4, 6, 12, 17]. Although both LAW and COW contain hydrogen peroxide, their slightly different concentrations did not result in a statistically significant difference in optical outcomes. In the present study, LAW ( ≈ 1.5% hydrogen peroxide) and COW ( ≈ 2.0% hydrogen peroxide) demonstrated comparable Δ*E*_00_ and ΔWID values across all materials, suggesting that the presence of hydrogen peroxide may be more influential than minor concentration variations under daily-use conditions. In contrast, the non-peroxide-based R.O.C.S. mouthrinse exhibited a limited preventive effect, which can be attributed to its reliance on mechanical or enzymatic surface-cleaning mechanisms rather than oxidative bleaching. Within the limitations of this in vitro model, these findings support the greater preventive efficacy of low-concentration peroxide-containing mouthrinses compared with non-peroxide formulations.

When the results were evaluated in relation to the chemical composition of the tested materials, composites with a nanohybrid structure and a more homogeneous filler distribution, such as ESQ, demonstrated greater optical stability across all parameters. This behavior can be attributed to the presence of uniformly sized spherical fillers and a well-matched refractive index between the resin matrix and filler particles, which reduces light scattering and limits pigment penetration. In contrast, bulk-fill and microhybrid composites, characterized by larger filler particles and modified resin matrices designed to enhance depth of cure, exhibited higher color changes, likely due to increased light scattering and greater susceptibility to extrinsic pigment adsorption. These findings indicate that filler morphology and matrix–filler compatibility play a more decisive role in optical stability than the mere presence of whitening agents.

In addition to their effects on restorative materials, the biological implications of whitening mouthrinses on natural enamel and oral soft tissues should be considered for proper clinical contextualization. Previous studies have demonstrated that low-concentration hydrogen peroxide–containing whitening products primarily exert a surface-limited oxidative effect, resulting in minimal and mostly reversible alterations in enamel microhardness and surface morphology, particularly under conditions simulating daily over-the-counter use [10, 19, 20]. Salivary buffering capacity and fluoride exposure have been reported to further mitigate these transient changes through remineralization processes. Regarding oral soft tissues, whitening mouthrinses containing low concentrations of hydrogen peroxide are generally well tolerated when used according to manufacturers’ instructions, with reported adverse effects limited to mild and temporary mucosal irritation in susceptible individuals [21, 24]. Non-peroxide–based whitening formulations, which act mainly through mechanical stain removal or enzymatic activity, have been associated with an even lower risk of mucosal irritation due to the absence of oxidative agents [14, 25]. Importantly, the present in vitro study did not aim to evaluate the biological effects of whitening mouthrinses on enamel or oral mucosa; therefore, these considerations are provided solely as supportive background information to contextualize the observed optical behavior of resin composites rather than as experimental outcomes.

The immersion and cycling regimen applied in this study constitutes an accelerated stress model intended to exaggerate staining and whitening effects within a limited time frame. Consequently, the protocol may represent a more intensive exposure than that typically encountered under routine clinical conditions. The main limitation of this study is that it was conducted in vitro. In the oral environment, factors such as saliva, pH fluctuations, thermal cycling, and mechanical abrasive forces may influence color stability. Moreover, only three composite materials and three whitening solutions were evaluated; therefore, assessing different filler systems and conducting longer-term evaluations would be valuable in future research. Further studies should include extended experimental designs incorporating thermal and mechanical aging protocols and should comparatively investigate the effects of varying peroxide concentrations.

## Conclusion

Whitening mouthrinses reduced coffee-induced color changes in composite resins under daily-use conditions. Hydrogen peroxide–containing formulations (LAW and COW) showed greater preventive efficacy than the non–peroxide-based mouthrinse (R.O.C.S.), although the observed whitening remained below the clinically perceptible threshold. The nanohybrid composite ESQ exhibited the most stable optical behavior across all parameters. Overall, hydrogen peroxide–based mouthrinses may help maintain the esthetic stability of composite restorations without adversely affecting translucency.

## Data Availability

All data included in this study are available upon request by contact with the corresponding author.
